# The cost-effectiveness of PHQ screening and collaborative care for depression in New York City

**DOI:** 10.1371/journal.pone.0184210

**Published:** 2017-08-31

**Authors:** Boshen Jiao, Zohn Rosen, Martine Bellanger, Gary Belkin, Peter Muennig

**Affiliations:** 1 Mailman School of Public Health, Columbia University, New York, NY, United States of America; 2 Ecole des Hautes Etudes en Santé Publique, 15 Avenue du Professeur Léon-Bernard—CS, Rennes, France; 3 New York City Department of Health and Mental Hygiene, Long Island City, NY, United States of America; University of Sheffield, UNITED KINGDOM

## Abstract

**Background:**

Depression is under-diagnosed and under-treated in most areas of the US. New York City is currently looking to close gaps in identifying and treating depression through the adoption of a screening and collaborative care model deployed throughout the city.

**Methods:**

We examine the cost-effectiveness of universal two-stage screening with the 2- and 9-item Patient Health Questionnaires (PHQ-2 and PHQ-9) in New York City followed by collaborative care for those who screen positive. We conducted microsimulations on hypothetical adult participants between ages 20 and 70.

**Results:**

The incremental cost-effectiveness of the interventions over the average lifespan of a 20-year-old adult in NYC is approximately $1,726/QALY gained (95% plausible interval: cost-saving, $10,594/QALY gained).

**Conclusions:**

Two-stage screening coupled with collaborative care for depression in the clinical setting appears to be significantly less expensive than most clinical preventive interventions, such as HIV screening in high-risk patients. However, effectiveness is dependent on the city’s ability to manage scale up of collaborative care models.

## Introduction

In New York City, ranks second, after ischemic heart disease, in terms of Disability Adjusted Life Years (DALYs) (one DALY is defined as one year of healthy life lost to disease) [1]. Moreover, depression takes a large economic toll in New York City, mostly in the form of health care costs and lost productivity in the workplace [1].

Because depression remains largely under-diagnosed and is treatable, improved screening and treatment could reduce the burden of disease due to depression. In New York City, only about half of the cases of adult depression are diagnosed in a clinical setting [2]. The rate of undiagnosed depression is obtained by screening samples of the city population with a sensitive and specific screening instrument, and then asking the participants whether they have ever been diagnosed with clinical depression. Routine primary care screening improves the diagnosis rates of adult depression, and has been recommended by the US Preventive Services Task Force (B recommendation) [3,4].

One inexpensive but also sensitive way to screen for depression is to use the 2-item Patient Health Questionnaire (PHQ-2) followed by the 9-item (PHQ-9) version for people who screen positive on the brief questionnaire. The PHQ is a well-validated screening tool [5]. The PHQ-2, which consists of the first two items of PHQ-9, can be used as a rapid screening test and has a high sensitivity, but low specificity [6]. By administering the remaining 7 questions, the specificity of the test improves, resulting in a reasonable “positive predictive value,” a measure of how often people are correctly diagnosed given the underlying prevalence of disease in the general population [7].

However, even when depression is diagnosed, it is often under-treated [8]. Adequate treatment is defined as receiving at least 8 psychotherapy visits or 4 medication monitoring visits within a year [9]. By this definition, less than half of those diagnosed with depression receive “adequate” treatment in the United States [9]. Collaborative care (CC) models have been promoted to address this problem [10]. In CC, a team approach is used to: 1) monitor the severity of depression, 2) provide proactive follow-up, and 3) ensure regular psychiatric consultation focused on treatment changes for patients who are not improving [11]. Randomized controlled trials have demonstrated that patients with depression who are being treated with CC have significantly greater adherence to treatment, more active adjustment of treatment in response to measured improvement, and better outcomes [12,13]. Of course, in the real world, CC teams probably vary significantly with respect to the quality, efficiency, and effectiveness of the care that they provide.

In 2015, the government of New York City announced ThriveNYC, a comprehensive strategy including 54 initiatives aimed at improving the mental health of New Yorkers [1]. As part of this plan, New York City committed to increasing the availability of screening and implementation of CC models. These models are to be adopted by primary care providers working in the “NYC Mental Health Corps,” an initiative that will engage approximately 400 professionals across the city [1]. At the same time, New York State, through reforms to its Medicaid program, is also promoting the growth of CC through the “1115 Medicaid waiver” which is a policy that in part provides incentives for adoption of this model in large health systems. The goal of the 1115 waiver is for 80% of New Yorkers having access to “advanced primary care”, which includes CC [14,15]. Central to both the city-level and state-level approaches are improved screening with the PHQ and improved treatment with CC. However, the program is a new investment, and is of unknown value.

We therefore project the cost-effectiveness of universal PHQ-2 screening followed by PHQ-9 and paired with CC for those who screen positive in New York City. We compared the interventions to “no screening” plus “usual care” as defined by the current mix of diagnosis and treatment in the community (the status quo).

## Method

### Overview and definitions

Our model calculates the proportion of true positives, false positives, true negatives, and false negatives using the sensitivity and specificity of the PHQ-2, the sensitivity and specificity of the PHQ-9, and the underlying prevalence of undiagnosed depression in New York City. Costs and outcomes are assigned to both correct and incorrect diagnoses relative to what would be expected to occur under the status quo in the real world (Model inputs can be found in Table 1).

**Table 1:** Values used in the Markov Model evaluating PHQ screening and collaborative care for adult depression in primary care of New York City versus the status quo.

Variable	Base	High	Low	Source
**Utility (HRQL score)**				
Depression	0.52	0.58	0.47	Mann et al. (2009)
Full remission achieved	0.76	0.82	0.7	Mann et al. (2009)
**Cost**				
*Direct cost*				
Treatment in collaborative care, $	2,879	3,599	2,159	Katon et al. (2002)
Treatment in usual care, $	2,016	2,520	1,512	Katon et al. (2002)
Screening, $	5	7	3	Estimated as above
*Indirect cost*				
Productivity, $	2,584	2,584	0	Greenberg et al. (2015)
**Probability**				
Treatable depression	0.67	0.71	0.63	Rush et al. (2006)
Diagnosis in status quo	0.52	0.62	0.42	NYC HANES 2013–2014
Treatment if diagnosed	0.61	0.74	0.48	NYC HANES 2013–2014
Adequate treatment in collaborative care	0.75	0.83	0.67	Katon et al. (2002)
Adequate treatment in usual care	0.44	0.53	0.35	Katon et al. (2002)
**Cut-off point of screening**				
PHQ-2	3	4	2	Kroenke et al. (2003)
PHQ-9	10	11	9	Kroenke et al. (2001)
*Corresponding sensitivities and specificities*				
Sensitivity, PHQ-2	83%	73%	93%	Kroenke et al. (2003)
Specificity, PHQ-2	90%	93%	74%	Kroenke et al. (2003)
Sensitivity, PHQ-9	88%	83%	95%	Kroenke et al. (2001)
Specificity, PHQ-9	88%	89%	84%	Kroenke et al. (2001)

The CC model in New York City is assumed to be equivalent in cost and efficacy to that in the literature, and to include: patient education, an initial two-session treatment with a psychiatrist in the primary clinic, a maximum of 3 months of shared care with the psychiatrist and primary care physician, and monitoring that assesses the need for follow-up visits and the adequacy of medication dose [13] (Model assumptions were all tested in sensitivity analyses, and can be found in Table 2).

**Table 2:** Assumptions used in the Markov Model evaluating PHQ screening and collaborative care for adult depression in primary care of New York City versus the status quo.

1. The HQRL scores associated with depression and remission were based on a randomized trial in the North of England. We assumed that they were generalizable to New York City.
2. HQRL score for those receiving response to treatment with no remission is difficult to be identified. It was therefore assumed that utility did not increase in those patients.
3.There are few data on HRQL score for healthy people receiving depression treatment. We assumed that there was no utility change in those people.
4. Since suicide and death by suicide are relatively rare, it was assumed that the suicide death caused by depression and the related cost would be negligible.
5. Most of probability estimates are based on a survey of general population. We assumed that they would remain the same in primary care.
6. The care models were derived from a randomized trial in Seattle. It was assumed that the results would keep the same in New York City.
7. There are few data on the risk ratio of depression among primary care patients to general population in New York City. We therefore approximated it using the ratio in the United States.

To assess the cost-effectiveness of the interventions, we built a Markov model using TreeAge Pro 2016. Our model estimated the costs and outcomes for the average New York City resident in primary care between the ages of 20 and 70. The quality-adjusted life year (QALY), defined as one year of perfect health, was used as an outcome measure. From a societal perspective, we included all costs, including screening costs and treatment costs, as well as indirect costs such as lost productivity. Time and transportation costs were not included, as they were deemed likely to be negligible relative to the screening and treatment costs and benefits. The indirect impact of the interventions cost reductions for other medical conditions was not included. Whether these gains are likely to be significant remains controversial, particularly among those with undiagnosed depression. For example, those with undiagnosed depression likely have less contact with the health care delivery system than those with diagnosed depression. Those who have developed co-morbid conditions are more likely to have had contact with a wide array of providers, and are therefore possibly more likely to have diagnosed depression. The extent to which treating depression reduces co-morbidity also remains controversial [16].

However, to the extent that screening and CC reduces co-morbidity, the health utility gain associated with depression comorbidities was at least partially captured in the measure that we use here, as it was based upon real-world cases of depression and depression remission [17]. To calculate the incremental cost-effectiveness ratio (ICER), we divided the additional money spent in the intervention arm by the additional gains in QALY. A 3% discount rate was used following recommendations of the Panel on Cost-effectiveness in Health and Medicine [18].

### Health utility

We used HRQL scores from a study on depression and depression remission that used the EuroQol questionnaire (EQ-5D) (see Table 1) [17]. The EQ-5D is comprised of 5 dimensions: mobility, self-care, usual activities, pain/discomfort and anxiety/depression. We assumed that there was no change in HRQL for patients without a remission.

### Costs

The monetary costs were adjusted to constant 2015 US dollars by using the Consumer Price Index of New York City [19,20]. The cost values included in our model are listed in Table 1 and the full cost breakdown is presented in S1 Table. The lost productivity associated with depression included the cost of absenteeism from the workplace, $609, and reduced productivity while at work, $1,975 [21]. It has been argued that the EQ-5D implicitly includes a measure of lost productivity [22]. Specifically, it controversially assumes that participants who engage in exercises meant to place a value on health are not only considering the effect of disability on their quality of life, but are also able to implicitly valuate the impact of their disease on their earnings and leisure time. This is especially problematic for a condition for which lost productivity is difficult to fathom [23]. For this reason, we analyzed the data both with and without these secondary measures of lost productivity. Additionally, lost productivity explicitly refers to production of goods and services. While cost-effectiveness standards suggest that lost productivity and leisure time be valued as equivalent, this may not reflect real-world valuations. A sensitivity analysis was therefore performed to apply lost productivity to participants aged 20–64 [24].

To estimate the screening cost, we assumed 1 minute of physician time would be required to review the PHQ-2 score, and 3 minutes for the PHQ-9., We assumed that a nurse would devote 6 minutes to administering this process [25,26]. These values reflect the mean time for screening, whether positive (which takes more time) or negative (which takes less time). We used the mean wages of a general practitioner and a registered nurse in New York City and adjusted for the probability of having positive or negative PHQ-2 test result based on the prevalence of depression and the sensitivity of the test. The mean hourly wages of general practitioners in New York City, $83, and registered nurses, $34, were obtained from United States Department of Labor [27].

The cost of CC includes the cost of antidepressant prescriptions and mental health and intervention visits; the cost of usual care has the same components minus the intervention visits [13].

### Probabilities

The age-specific probability of death was derived from a US life table (see S2 Table) [28]. We obtained the 12-month prevalence of depression from the New York City Health and Nutrition Examination Survey (NYC HANES) 2013–2014 (see S2 Table) [29]. It should be noted that the prevalence of depression in primary care was estimated to be 1.74 times higher than in general population of the United States [30,31]. Thus, to estimate the age-specific probabilities of depression, we multiplied the prevalence of depression in each age group by this number.

Additional model probability inputs are listed in Table 1 below. The probability of treatable depression was obtained from a Sequenced Treatment Alternatives to Relieve Depression (STAR*D) trial in which a cumulative remission rate of 67% was reported [32].

Scores of 3 and 10 are often recommended as the optimal cut-off points for a depression diagnosis within the PHQ-2 and PHQ-9 respectively [33–35]. Given these cut-off points, the sensitivity and specificity of the PHQ-2 for diagnosing depression were 88% and 88%, respectively; for the PHQ-9, they were 83% and 90%, respectively [33,34].

### Decision analysis models

**Fig 1:**
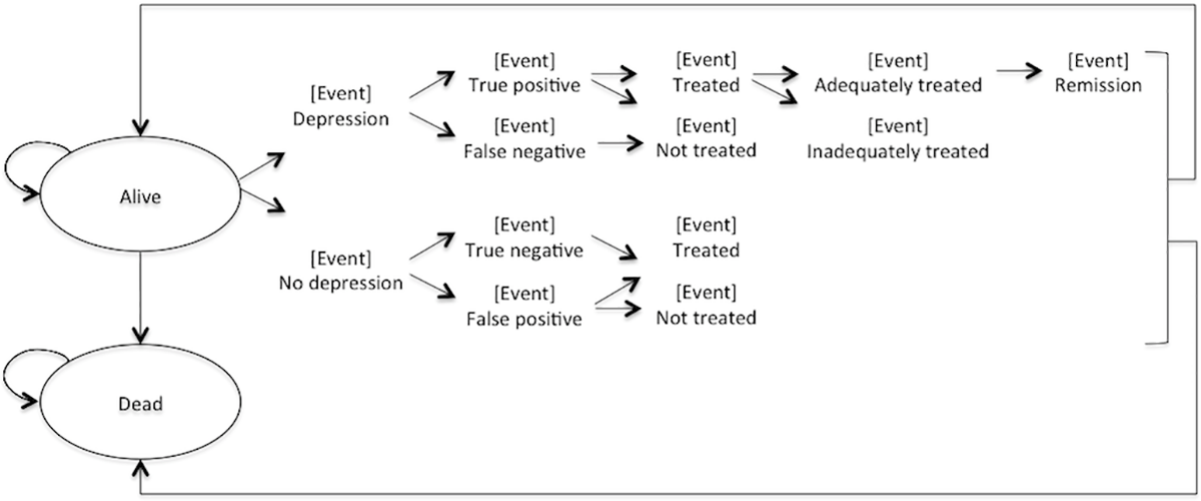
Model diagram.

The model incorporated two health states: “alive” and “dead”. Alive patients could be depressed or well, and depressed patients could be treated with CC or untreated [36]. The model diagram is presented in Fig 1, and the key assumptions are listed in Table 2. In our model, we estimate the costs and QALYs associated with the status quo versus universal PHQ-2 screening, followed by PHQ-9 screening for positive screens and CC [36]. The status quo arm includes all costs and benefits currently realized from the availability of primary and psychiatric care in the community. This includes costs and QALYs gained from lost productivity, current care seeking behaviors, and treatment programs. Participants in the status quo arm are exposed to his or her age-specific probability of depression and death, and include patients who are both treated and untreated for depression. Participants who “die” exit the model and simulated participants who “survive” exposure to this probability of death remain in the model.

The universal screening and treatment arm includes an estimate of the increased benefits from having a formal screening program in place alongside a CC model (thereby somewhat improving both the detection and management of clinical depression). Moreover, the probability of depression is reduced by the incremental remission rate of the previous life cycle (both because early treatment may reduce relapse and because CC involves a broader range of modalities for resistant depression).

### Sensitivity analyses

We conducted a series of one-way sensitivity analyses along with a Monte Carlo simulation. In the Monte Carlo simulation, a triangular distribution was used for every variable in the model. The random error associated with an estimate or plausible boundaries for the values were included in this distribution.

## Results

The results of the fully incremental analysis are presented in Table 3. If $ 40,000 per QALY was chosen as a conservative threshold of willingness-to-pay, two-stage screening followed by CC was more cost-effective than any other strategy such as screening with PHQ-9 only or screening without CC. Implementing two-stage depression screening and CC in primary care in New York City increases the discounted societal costs by $660 per person over a 50-year interval from age 20 to 70. The average 20-year-old would gain 0.38 QALY over this interval, resulting in an ICER of $1,726/QALY at a discount rate of 3% (Table 3, values rounded).

**Table 3:** Costs (in 2015 US dollars), incremental cost, quality-adjusted life years (QALYs) gained, incremental QALYs gained, and incremental cost-effectiveness (ICER) of screening and collaborative care for adult depression in primary care of New York City versus the Status Quo (A fully incremental analysis).

Strategy	Cost, $	Incremental cost, $	Effectiveness, QALY	Incremental effectiveness	ICER, $
1. No screening; Usual care	11,867		24.19		
2. PHQ 2/9 screening; Usual care	12,922	1,055	24.31	0.12	Extendedly dominated by 1&4
3. PHQ 9 screening; Usual care	15,830	2,908	24.37	0.06	Extendedly dominated by 1&4
**4.PHQ2/9 screening; Collaborative care** ^**a**^	**12,528**	**660**	**24.57**	**0.38**	**1,726**
5. PHQ 9 screening; Collaborative care ^b^	16,537	4,009	24.65	0.08	50,113

a versus Strategy 1

b versus Strategy 4

Table 4 lists the effects of a series of one-way sensitivity analyses on ICERs. The treatment cost associated with CC produced more variance in the ICER than any other cost variable. Likewise, the probability of the adequacy of CC treatment was the most influential probability input. If we assumed that the EQ-5D included a measure of lost productivity and withdrew the associated cost, the ICER would increase to $8,840/QALY. If we only applied lost productivity to the working age population (thereby removing monetary valuation of leisure time losses), an ICER of $1,870/QALY would be obtained.

**Table 4:** One-way sensitivity analyses of variables Included in the model.

	Incremental Cost, $		Incremental Effectiveness, QALY		ICER	
Variable	High	Low	High	Low	High	Low
Treatment cost in CC ^a^	2,110	790	0.38	0.38	5,515	Dominance
Lost productivity 1 ^b^	660	3,383	0.38	0.38	1,726	8,840
Lost productivity 2 ^c^	660	716	0.38	0.38	1,726	1,870
Probability of adequate treatment in CC	100	1,234	0.44	0.32	226	3,850
Cut-off point of PHQ-2	705	1,227	0.33	0.43	2,113	2,843
Cut-off point of PHQ-9	719	678	0.36	0.42	2,004	1,631
Probability of depression ^d^	656	662	0.47	0.31	1,396	2,112
Probability of treatment if diagnosed	667	617	0.45	0.31	1,471	1,997

a CC = collaborative care

b High value: Lost productivity = $2,584; Low value: Lost productivity = 0

c High value: Lost productivity for all the hypothetical participants; Low value: Lost productivity for those aged 20–64

d The plausible range is ± 20% of the base.

**Fig 2:**
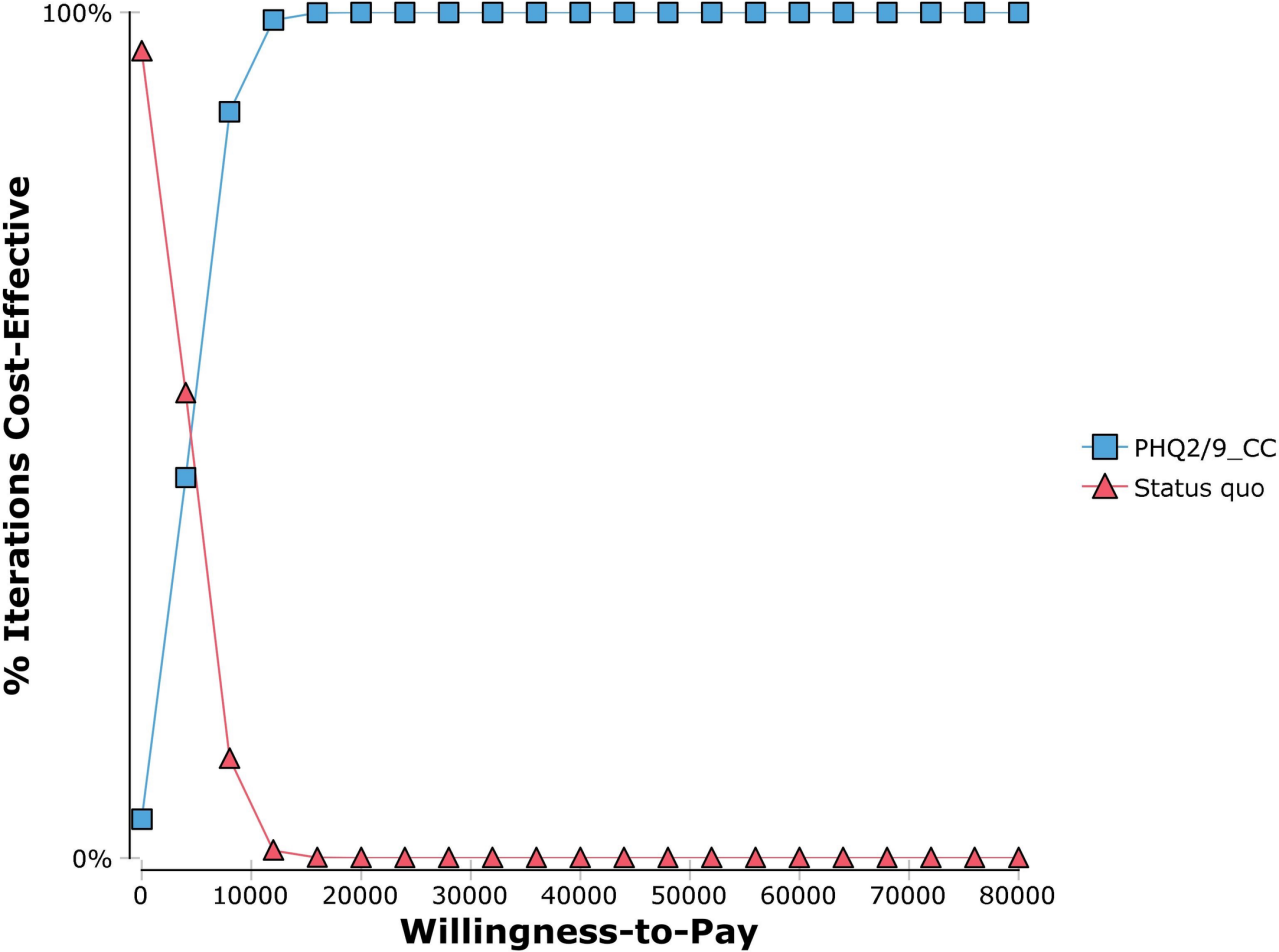
Cost-effectiveness acceptability curve, PHQ screening and collaborative care for adult depression in primary care of New York City versus the Status Quo.

**Fig 3:**
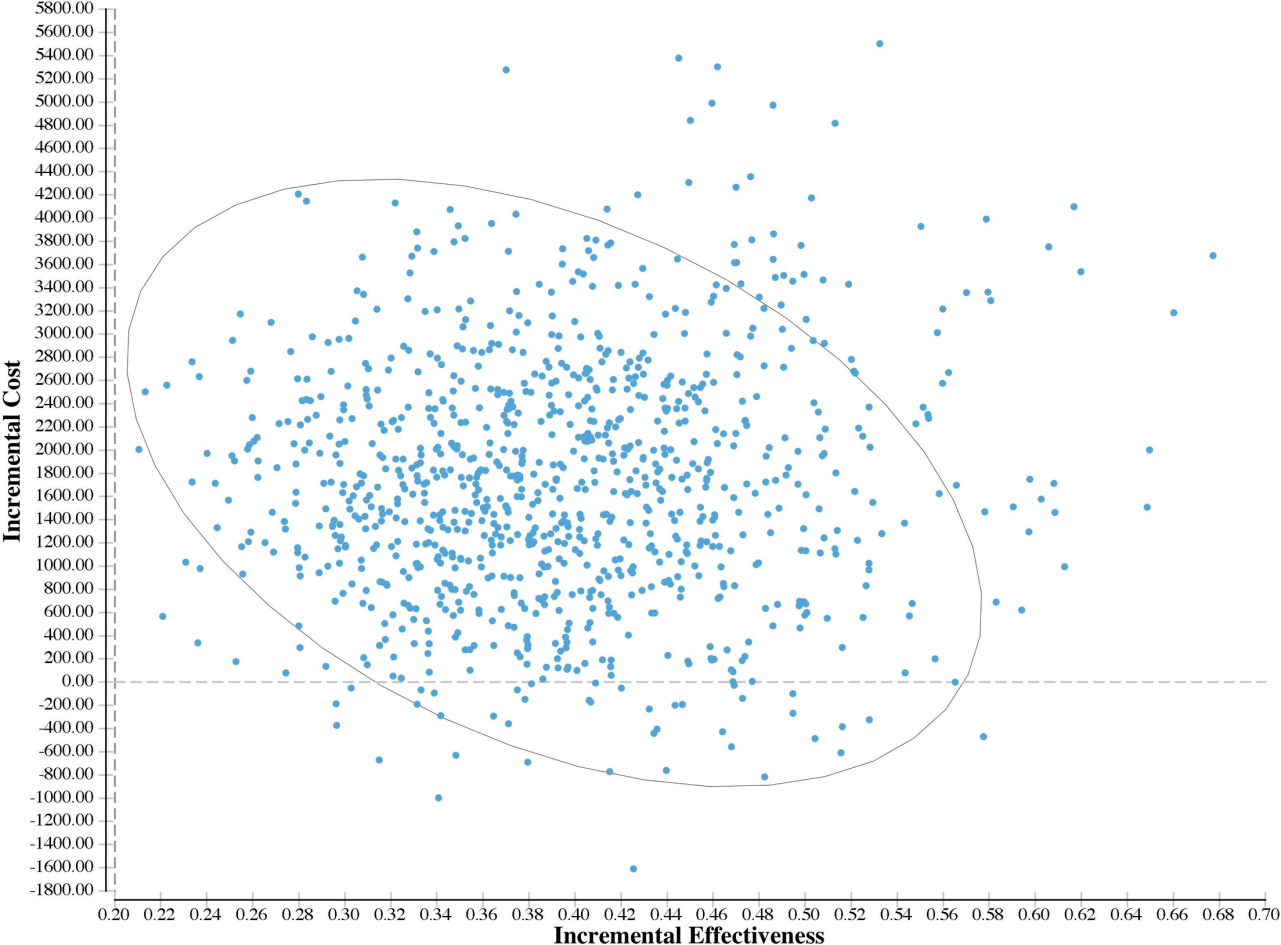
Incremental cost-effectiveness scatter plot, PHQ screening and collaborative care for adult depression in primary care of New York City versus the Status Quo.

There was an increase in the ICER when lowering the cut-off score for the PHQ-2. Conversely, lowering the cut-off score of PHQ-9 decreased the ICER. In the Monte Carlo analysis, the 95% plausible interval of the ICER ranged from dominance (where treatment actually saves money overall) to $10,594/QALY. (By convention, the word plausible is used to highlight the difference in estimation used between traditional confidence intervals based on random error and error generated by simulation, which includes both random and “plausible” non-random error). The cost-effectiveness acceptability curve and the incremental cost-effectiveness scatter plots are presented in Figs 2 and 3 respectively. Additionally, a graph showing the relationship between the expected value of perfect information versus willingness-to-pay is shown in S1 Fig.

## Discussion

Randomized controlled trials (RCTs) have shown that screening for depression in the primary care setting generally increases the diagnosis of depression [3]. Similarly, RCTs have shown that CC improves treatment adequacy [12,13]. Taken together, substantial gains in depression remission rates could be achieved with universal screening and CC in clinical settings. We found that the quality-adjusted life expectancy of those who receive these interventions would increase by about 0.38 QALYs relative to those who do not. This increase is substantial, coming close to the gains associated with smoking cessation [37].

Depression screening itself consumes valuable practitioner time, and the cost of CC is about $860 more costly per patient compared to usual care. Nevertheless, the average incremental net cost across the entire life course (with remissions and exacerbations) was only $660. This is mainly because a considerable amount of lost workplace productivity associated with depression would be saved if remission were achieved. In the United States, the greatest direct economic loss linked to depression is lost productivity [21]. Remission also reduces costs associated with future medical treatments. However, the healthy may be misdiagnosed when there are false positives (a particular problem in low-prevalence settings). Such patients not only spend time in treatment and incur the cost of medication and provider time, they may also experience side effects associated with medications. Similar concerns apply to those who have depression, but who do not respond to treatment.

Indeed, we find that it may be cost-effective to raise the cut-off point of the PHQ-2 so that fewer false positives occur. The PHQ-2 has modest specificity but very high sensitivity [6,34]. Lowering the threshold for detection, and thus classifying more patients as in need of treatment [6,34], will both increase program costs associated with administering the PHQ-9 and costs associated with treating people without depression. Hence, raising the cut-off point of the PHQ-2 seems be a more cost-effective option than doing so for the PHQ-9. Ultimately, though, the cut-off points depend on the policy maker’s view of the trade-off between cost and effectiveness.

This study is prone to a number of important limitations. First, the probability of treatment adequacy in CC and the health utility estimates were derived from randomized trials in Seattle and Northern England respectively, which might not be generalizable to New York City. It is also important to remember that additional various and complex barriers, such as stigma and financial cost, affect many patients’ ability to continue treatment for their depression [38], and these considerations were not included. Another limitation of the study is that we did not calculate the changes in QALYs for those who do not achieve remission but who did respond partially to the treatments. Depressive symptoms may be reduced in this group of patients [32], but there is, to date, inadequate information on partial response rates. Inclusion of such values could increase the cost-effectiveness of the interventions.

In our study, the background 12-month prevalence of depression was assessed using the World Health Organization Composite International Diagnostic Interview (CIDI), which was assumed to be the gold standard. However, the diagnosis of depression is complex, and no single instrument is 100% sensitive and specific. Additionally, our probabilistic sensitivity analysis—a Monte Carlo simulation with triangular distributions—does not account for many nuances in the data. Within each distribution, we included random error in addition to a mutually-agreed upon estimation of non-random error with somewhat arbitrary endpoints. While sampling many triangular distributions will produce a normal distribution, the final distribution is likely to contain much wider confidence intervals than many other approaches. Finally, some inputs of the model, such as the probability of depression diagnosis and treatment, were derived from a general population survey in New York City. It is likely that different probability estimates would be achieved if studied specifically within the primary care settings. For example, there is evidence that white Americans have more access to primary care, and are also more likely to receive treatment for depression than non-white Americans [39,40]. Values may differ depending on the population to which the interventions are applied. Moreover, as the prevalence of undiagnosed depression declines in any given population, the positive predictive value screening declines alongside the ICER.

More medical costs would be saved and more effectiveness would be gained if depression comorbidity were included as costs in our analysis. Even without it, however, our models suggest that PHQ screening coupled with CC appear to be very cost-effective.

We model the two interventions that have received the most attention in the literature. ThriveNYC, an ambitious mental health plan in New York City, consists of a wide variety of initiatives, including extending opportunities for screening, outside of clinical settings altogether. Such approaches could reach even higher risk populations and higher need communities, groups that underutilize primary care [1], but our models do not include such approaches. It is not appropriate to generalize our findings to settings in which the prevalence of undiagnosed depression may be lower than those we observe in clinical settings.

ThriveNYC is also concerned with large scale public messaging and other initiatives to change the current culture around mental health, and specific initiatives have been developed to reframe people’s perception about mental health [1]. Screening and CC can provide even greater value if stigma, a significant barrier to depression treatment, is reduced [41]. However, these broader programs are of unknown efficacy and may increase costs without brining additional benefits.

Nevertheless, by expanding our models to broader, integrated initiatives, it may be possible to find synergies that produce even greater social benefits that we observe here. Our models can support smarter public health approaches for mental health, as starting points for the inclusion of cost-effectiveness goal-setting in policymaking.

## Supporting information

S1 Fig The relationship between expected value of perfect information (EVPI, y-axis) and willingness to pay (x-axis) in our cost-effectiveness model. (TIF)

S1 Table Complete list of costs used in our analysis. **All costs were adjusted to constant 2016 dollars.** ^a^ Source: Arias E. United States life tables, 2011. Natl Vital Stat Rep [Internet]. 2015 Sep;64(11). Available from: http://www.cdc.gov/nchs/data/nvsr/nvsr64/nvsr64_11.pdf.
^b^ Source: New York City Department of Health and Mental Hygiene. 2013–2014 New York City Health and Nutrition Examination Survey. 2015.
(DOCX)

S2 Table Age-specific probabilities of depression and of death used in our analysis. (DOCX)
