# Supplementary figures and images for: The cost-effectiveness of PHQ screening and collaborative care for depression in New York City
Source: PLoS One. 2017 Aug 31;12(8):e0184210. doi: 10.1371/journal.pone.0184210 (PMC5578679; doi:10.1371/journal.pone.0184210)

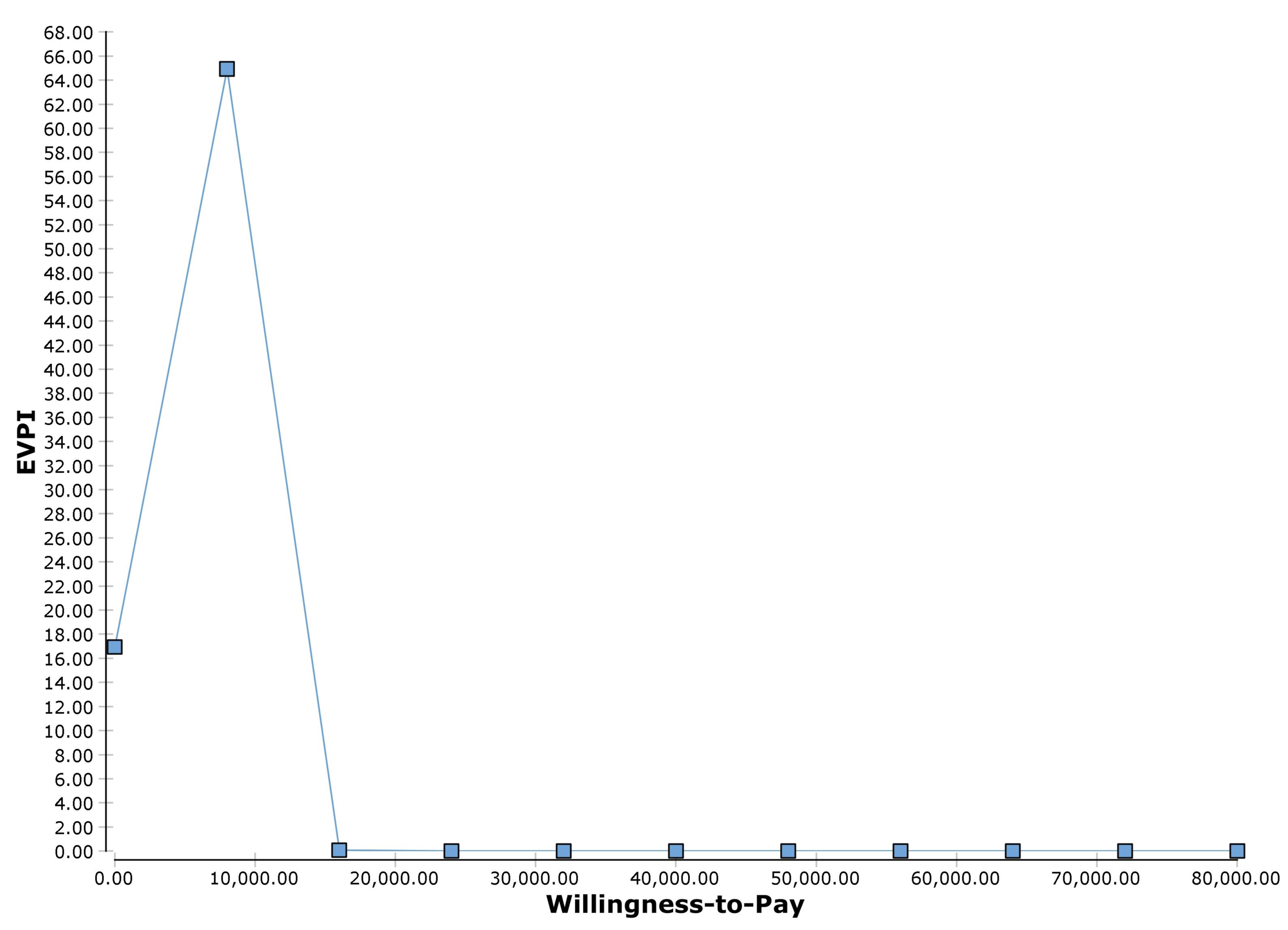

Supplement: S1 Fig — (TIF) [file pone.0184210.s001.tif]
