# Supplementary material for: The cost-effectiveness of PHQ screening and collaborative care for depression in New York City
Source: PLoS One. 2017 Aug 31;12(8):e0184210. doi: 10.1371/journal.pone.0184210 (PMC5578679; doi:10.1371/journal.pone.0184210)
Supplement: S1 Table — All costs were adjusted to constant 2016 dollars. a Source: Arias E. United States life tables, 2011. Natl Vital Stat Rep [Internet]. 2015 Sep;64(11). Available from: http://www.cdc.gov/nchs/data/nvsr/nvsr64/nvsr64_11.pdf. b Source: New York City Department of Health and Mental Hygiene. 2013–2014 New York City Health and Nutrition Examination Survey. 2015. (DOCX) [file pone.0184210.s002.docx]

| **Item** | **Cost $** | **Source** |
| --- | --- | --- |
| ***Treatment in collaborative care*** |  |  |
| Antidepressant prescriptions | 1152 | Katon et al. (2002) |
| Specialty mental health visits | 864 | Katon et al. (2002) |
| ***Treatment in usual care*** |  |  |
| Antidepressant prescriptions | 1961 | Katon et al. (2002) |
| Specialty mental health visits | 658 | Katon et al. (2002) |
| Intervention visits | 260 | Katon et al. (2002) |
| ***Screening cost*** |  |  |
| PHQ-2, physician time cost | 1 | Smarr et al. (2011) |
| PHQ-2, nurse time cost | 3 | Valenstein et al. (2001) |
| PHQ-9, physician time cost | 4 | Smarr et al. (2011) |
| PHQ-9, nurse time cost | 3 | Valenstein et al. (2001) |
| ***Lost productivity*** |  |  |
| Absenteeism | 609 | Greenberg et al. (2015) |
| Presenteeism | 1975 | Greenberg et al. (2015) |
