# Supplementary material for: The cost-effectiveness of PHQ screening and collaborative care for depression in New York City
Source: PLoS One. 2017 Aug 31;12(8):e0184210. doi: 10.1371/journal.pone.0184210 (PMC5578679; doi:10.1371/journal.pone.0184210)
Supplement: S2 Table — (DOCX) [file pone.0184210.s003.docx]

| **Age** | **% Death ^a^** | **% Depression (12-month prevalence) ^b^** |
| --- | --- | --- |
| 20 | 0.074% | 9.0% |
| 21 | 0.083% |  |
| 22 | 0.089% |  |
| 23 | 0.092% |  |
| 24 | 0.093% |  |
| 25 | 0.093% | 12.0% |
| 26 | 0.094% |  |
| 27 | 0.096% |  |
| 28 | 0.098% |  |
| 29 | 0.102% |  |
| 30 | 0.106% | 9.6% |
| 31 | 0.109% |  |
| 32 | 0.113% |  |
| 33 | 0.117% |  |
| 34 | 0.120% |  |
| 35 | 0.125% | 5.6% |
| 36 | 0.131% |  |
| 37 | 0.139% |  |
| 38 | 0.148% |  |
| 39 | 0.158% |  |
| 40 | 0.169% | 3.3% |
| 41 | 0.181% |  |
| 42 | 0.197% |  |
| 43 | 0.217% |  |
| 44 | 0.241% |  |
| 45 | 0.265% | 10.7% |
| 46 | 0.291% |  |
| 47 | 0.320% |  |
| 48 | 0.351% |  |
| 49 | 0.385% |  |
| 50 | 0.420% | 11.7% |
| 51 | 0.456% |  |
| 52 | 0.493% |  |
| 53 | 0.530% |  |
| 54 | 0.570% |  |
| 55 | 0.613% | 7.1% |
| 56 | 0.660% |  |
| 57 | 0.710% |  |
| 58 | 0.762% |  |
| 59 | 0.816% |  |
| 60 | 0.873% | 13.2% |
| 61 | 0.934% |  |
| 62 | 0.998% |  |
| 63 | 1.072% |  |
| 64 | 1.157% |  |
| 65 | 1.259% | 3.9% |
| 66 | 1.376% |  |
| 67 | 1.506% |  |
| 68 | 1.638% |  |
| 69 | 1.776% |  |
| 70 | 1.930% |  |
